# Yeast-Host Interactions: *Anadenanthera colubrina* Modulates Virulence Factors of *C. albicans* and Inflammatory Response *In Vitro*

**DOI:** 10.3389/fphar.2021.629778

**Published:** 2021-06-08

**Authors:** Carolina Medeiros de Almeida Maia, Silvana Pasetto, Cassiano Francisco Weege Nonaka, Edja Maria Melo de Brito Costa, Ramiro Mendonça Murata

**Affiliations:** ^1^ Department of Dentistry, Postgraduate Program in Dentistry, State University of Paraiba, Campina Grande, Brazil; ^2^ Department of Foundational Sciences, School of Dental Medicine, East Carolina University, Greenville, NC, United States

**Keywords:** phytotherapy, antifungal agents, oral candidiasis, *Candida albicans*, biofilm, virulence factors, immune response

## Abstract

Oral candidiasis is one of the most common fungal infections in humans. Its incidence has increased widely, as well as the antifungal resistance, demanding for the search for novel antifungal therapeutic agents. *Anadenanthera colubrina* (Vell.) Brenan is a plant species that has been proven to possess pharmacological effects, including antifungal and anti-inflammatory activities. This study evaluated *in vitro* the effects of standardized *A. colubrina* extract on virulence factors of *Candida albicans* and its regulation on immune response through *C. albicans*-host interaction. Antifungal activity was evaluated by Broth Microdilution Method against reference *Candida* strains (*C. albicans*, *C. glabrata*, *C. tropicalis*; *C. dubliniensis*). Anti-biofilm effect was performed on *C. albicans* mature biofilm and quantified by CFU/mL/g of biofilm dry weight. Proleotlytic enzymatic activities of proteinase and phospholipase were assessed by Azocasein and Phosphatidylcholine assays, respectively. Cytotoxicity effect was determined by Cell Titer Blue Viability Assay on Human Gingival Fibroblasts. Co-cultured model was used to analyze *C. albicans* coexisting with HGF by Scanning Electron Microscopy and fluorescence microscopies; gene expression was assessed by RT-PCR of *C. albicans* enzymes (SAP-1, PLB-1) and of host inflammatory cytokines (IL-6, IL-8, IL-1β, IL-10). Cytokines secretion was analysed by Luminex. The extract presented antifungal effect with MIC<15.62 μg/ml against *Candida* strains. Biofilm and proteolytic activity were significant reduced at 312.4 μg/ml (20 × 15.62 μg/ml) extract concentration. Cell viability was maintained higher than 70% in concentrations up to 250 μg/ml (LD_50_ = 423.3 μg/ml). Co-culture microscopies demonstrated a substantial decreased in *C. albicans* growth and minimal toxicity against host cells. Gene expressions of SAP-1/PLB-1 were significantly down-regulated and host immune response was modulated by a significant decreased on IL-6 and IL-8 cytokines secretion. *A. colubrina* had antifungal activity on *Candida* strains, antibiofilm, and anti-proteolytic enzyme effects against *C. albicans*. Presented low cytotoxicity to the host cells and modulatory effects on the host immune response.

## Introduction

Oral candidiasis is one of the most common fungal infections in humans (Hertel et al., 2016; Rosa-García et al., 2020) and is caused by yeasts from the genus *Candida* (Williams and Lewis, 2011), a polymorphic fungus and a commensal microorganism that colonizes the human oral cavity in healthy people (Nikou et al., 2019). However, under circumstances where host immunity is impaired, *Candida* spp. can switch its harmless phenotype to a pathogenic form capable of breaching mucosal barriers (Dantas et al., 2016), causing from superficial mucosal infection to deep seated invasive and life-threatening disseminated disease (Lewis and Williams, 2017; Nikou et al., 2019), which is related to a high mortality rate (58–81%) (Vaezi et al., 2017).

*Candida albicans* is the species most often associated to oral candidiasis (Prieto et al., 2016; Pappas et al., 2018), accounting for up to 95% of the cases (Vila et al., 2020). Its overgrowth and invasion of superficial tissues is dependent on the host’s defenses and the virulence factors of the fungus (Tooyama et al., 2015; Millsop and Faze, 2016; Hellstein and Marek, 2019), such as, adherence to oral epithelial or medical devices surfaces; biofilm formation; destruction of host tissue through secretion of proteolytic enzymes; evasion of host defenses invasion mechanisms and development of drug resistance (Höfs et al., 2016; Vila et al., 2020).

Considering the continuing rise of resistant *Candida* spp. strains and the limited number of antifungal agents, novel therapeutic strategies have been directed toward the identification of bioactive compounds that target virulence factors and pathogenic mechanisms to prevent *C. albicans* transition from harmless commensal to pathogen (Francisconi et al., 2020; Vila et al., 2020).

In this regard, natural products from plants are considered a potential source for the development of new antifungal therapies. Between the years of 1940 and 2014, 40% of all molecules accepted by FDA (US Food and Drug Administration) were natural products (Newman and Cragg, 2016). *Anadenanthera colubrina* (Vell.) Brenan, popularly known as Angico, is a plant species that can be found in Brazil, from the Northeastern to the Southeastern regions. It is a woody species typical of the Caatinga Brazilian biome (Silva et al., 2019) and its use by the traditional communities of Brazilian semiarid as a medicinal plant is common, which includes the treatment of inflammation in general (Araújo et al., 2014; Araújo et al., 2015). Recent researches have shown that *A. colubrina* has promising therapeutic properties, such as antifungal (Lima et al., 2014; Silva et al., 2019), anti-proliferative (Lima et al., 2014), anti-inflammatory (Guarneire et al., 2019; Cardoso-Junior et al., 2020), antioxidant (Araújo et al., 2019; Cardoso-Junior et al., 2020), and anti-HIV (Maia et al., 2021).

Some studies regarding the antifungal effects of *A. colubrina* have suggested an inhibitory activity among *Candida* species, mainly on *C. albicans* biofilms. (Lima et al., 2014; Silva et al., 2019). Lima et al. (2014) demonstrated *in vitro* the strong antifungal activity of *A. colubrina* extract against *C. albicans* in planktonic culture and also its potential in inhibit the formation of *C. albicans* biofilm. Therefore, *A. colubrina* extract toxicity and therapeutic action were evaluated *in vivo* on *Galleria mellonella* model, having not affected the viability of the larvae at doses below 100 mg/kg and high potential for the treatment of *C. albicans* infection (Silva et al., 2019). These recent findings suggest that *A. colubrina* extract is a strong *Candida*te for development of a new agent for the treatment of oral candidiasis.

Despite this therapeutic potential, there is little information available about how this plant species can regulate the expression of virulence factors of *Candida* co-cultured with humans cells and the host immune response during the fungal infection as well. Therefore, the present study aimed to evaluate *in vitro* the modulatory effects of *A. colubrina* extract on major virulence factors related to the pathogenicity of *C. albicans* infection through the interaction between host and pathogen.

## Material and Methods

### Plant Material and Standardized Extraction Procedures

The plant material was collected during the month of September in the semi-arid region of Paraíba state, Brazil (7° 22′ 25″ S, 35° 59′ 32″ W). Botanical specimens of *Anadenanthera colubrina* (Vell.) Brenan were deposited in the Manuel de Arruda Câmara Herbarium (ACAM) at the State University of Paraíba (UEPB), Campus I, Campina Grande, Paraíba, Brazil, under nº 1936/ACAM. This research was conducted under authorization number SisGen A289DF4. A hydroethanolic standardized extract was obtained according to the method described by Carvalho et al. (2011). Briefly, hydroethanolic extract (80%, v/v) of the plant bark was obtained by maceration for 48 h using the proportion of 10 mg of the plant for each 25 ml of 80% ethyl alcohol. Three filtrations of the material were performed, followed by vacuum concentration (Tecnal TE-211, Piracicaba, SP) and lyophilization (Martin Christ 1-2 LDplus, Germany). An extraction yield of 31.7% was obtained.

Considering the same standardized extract (same batch of plant material and same extraction process) used in this research and used by Lima et al. (2014), Rocha et al. (2017), Lima et al. (2018–*data not published*) and Silva et al. (2019), the presence of polyphenol, tannins and flavonoids, compounds with straight relationship with *A. colubrina* extract pharmacological activity, were also recently monitored by gas chromatograph (GC) coupled to mass spectrometer (MS) with electron impact ionization (EI) (model GCMS-QP2010 Ultra, Shimadzu) according to the method described by Maia et al. (2021), using the same standardized material.

### Susceptibility Test

The antimicrobial activity of *A. colubrina* extract was assessed by Broth Microdilution Method against the following *Candida* spp: *C. albicans* ATCC^®^ 90028, *C. albicans* ATCC^®^ MYA-2876, *C. glabrata* ATCC^®^ MYA-275, *C. tropicalis* ATCC^®^ MYA-750; *C. dubliniensis* ATCC^®^ MYA-646, with the determination of the minimum inhibitory concentration (MIC) and minimum fungicidal concentration (MFC), according to CLSI guidelines (M27-A2) (Clinical and Laboratory Standards Institute (CLSI), 2002). The assay was performed in 96 well-plates (Greiner Bio-One North America, Inc. Monroe, NC) containing 100 µL/well of RPMI-1640 culture medium (Lonza Bioscience, Walkersville, MD). One hundred microliters of the extract were added to the initial well (8,000 μg/ml), followed by serial microdilution, obtaining concentrations between 2,000 and 15.62 μg/ml. Inoculum concentration was standardized using a spectrophotomer (SpectraMax M3, Molecular Devices, Sunnyvale, CA), by first measuring the absorbance in the range of 0.08–0.1 at 625 nm, which yielded a yeast stock solution equivalent to 5 × 10^6^ CFU/mL that was then diluted in RPMI-1640 medium to a final concentration of 5 × 10^3^ CFU/mL. Next, 100 μL of yeast suspension was added to each well, resulting in a final concentration of 2.5 × 10^3^ CFU/ml. The plates were incubated for 24 h at 37°C in 5% CO_2_ (VWR Symphony 5.3 A, Radnor, PA). Fluconazole (512 μg/ml) (Alfa Aesar^®^, Tewksbury, MA) was used as positive control. The vehicle control used was Dimethyl Sulfoxide 1% (DMSO, BDH Solvents, Dawsonville, GA). The MIC was defined as the lowest concentration of the sample capable of inhibiting visible microbial growth, as confirmed by the change in the color of the RPMI-1640 medium. For the determination of the MFC, an aliquot of 10 μL from each well with concentrations equal to or higher than the MIC was sub-cultivated in Sabouraud Dextrose Agar medium (BD Difco, Franklin Lakes, NJ) and incubated at 37°C, in 5% CO_2_, for 48 h. The MFC was defined as the smallest concentration that inhibited visible growth on the agar plates. All of the assays were performed in triplicates and repeated at least three different times for reproducibility (Seleem et al., 2016b).

### Biofilm Assay

An inoculum of 1 × 10^6^ CFU/mL of *C. albicans* (ATCC^®^ MYA-2876) was grown for 24 h in a sterile 24-well plate (Greiner Bio-One North America, Inc. Monroe, NC, United States) using Yeast Nitrogen Base Medium (YNB, Sigma Aldrich, San Luis, MO) with 50 mM of glucose (VWR Life Science, Radnor, PA) for 24 h at 37°C in 5% CO_2_ to establish initial biofilm growth. Total volume of 1 ml of inoculum was pipetted in each well. After 24 h of incubation, the biofilms were treated once daily with 100 µL of *A. colubrina* extract at concentrations equivalents to 156.2 μg/ml (10 × 15.62 μg/ml) and 312.4 μg/ml (20 × 15.62 μg/ml), which remained incubated with the biofilm suspended in medium overnight. The vehicle control used was 1% DMSO, while positive control was Fluconazole (10xMIC). Before each treatment, biofilms were washed with Phosphate Buffer Solution (PBS, Lonza Bioscience, Walkersville, MD) and replenished with 900 µL of fresh YNB medium in addition to 100 µL of the corresponding treatment, yielding a total volume of 1 ml in each well. After 72 h of treatments, adhered biofilms were collected by scraping the bottom of each well plate and suspending in PBS, which was then centrifuged at 10,000 rpm for 5 min. Biomass (dry weight) of each biofilm sample was obtained by discarding the supernatant and placing the samples in a speed vacuum to dry for 40 min. Colony formation unit (CFU) was determined by submitting the bioifilm suspension to serial dilutions (10^−1^, 10^−2^, 10^−3^, 10^−4^) and plating 10 µL of these dilutions on Sabouraud Dextrose Agar plates, which were incubated at 37°C in 5% CO_2_. After 24 h of incubation, the number of *C. albicans* colonies was counted and the data was normalized based on the CFU/ml/dry weight of biofilm sample (Santana et al., 2013; Seleem et al., 2016a; Seleem et al., 2016b; Chen et al., 2018).

### Proteinase and Phospholipase Enzyme Secretion Assay

Proteinase and phospholipase enzyme secretion assays were conducted as previously performed by Santana et al. (2013), Chen et al. (2018). Biofilms of *C. albicans* were grown for 24 h in YNB Medium with 50 mM of glucose at 37°C in 5% CO_2_ and treated with *A. colubrina* extract (156.2 μg/ml–10 × 15.62 μg/ml and 312.4 μg/ml–20 × 15.62 μg/ml). Trypsin (Gibco, Invitrogen) was used as standard. The vehicle control used was 1% DMSO. After 72 h of biofilm maturation, the enzyme secretion assays were performed on biofilms suspended in PBS, which were sonicated for 15 s at 20% amplitude with pulses at 5 s and 10 s intervals (FB120; Fischer Scientific, Pittsburgh, PA, United States). The proteinase enzyme activity was determined by mixing the supernatant of the biofilm solution with 1% azocasein (Sigma Aldrich, San Luis, MO) at 9:1 (v/v) for 1 h at 37°C in 5% CO_2_. Then, 500 µL of 10% trichloroacetic acid (VWR) was added to stop the reaction. The solution was centrifuged for 5 min at 10,000 rpm and 500 µL of the supernatant was combined with 500 µL of 0.5 M NaOH (Macron Fine Chemicals, Avantor VWR Life Science, Radnor, PA), which was incubated for 15 min at 37°C in 5% CO_2_. Absorbance was read in a spectrophotometer at 440 nm (Pande et al., 2006; Gonçalves et al., 2012; Santana et al., 2013; Chen et al., 2018). The phospholipase enzyme activity was determined by mixing the supernatant of the biofilm solution (pH corrected to 7.5) with phosphatidylcholine substrate (Sigma Aldrich, San Luis, MO) at 9:1 (v/v) for 1 h at 37°C in 5% CO_2_ and reading the absorbance in a spectrophotometer at 630 nm (Taniguchi et al., 2009). The rates of absorbance shifts (ΔOD) for the repetitions were adjusted by the blank. One enzyme unity was arbitrarily established as the absorbance shift, by minute of reaction, by biomass, multiplied by one thousand (*U* = ΔODnm × min−1 × 1,000). The specific enzyme activity was defined as the amount of enzyme that elicited an increase of 0.001 units of absorbance/minute of digestion by biofilm dry weight (g) (Taniguchi et al., 2009; Santana et al., 2013; Chen et al., 2018).

### Cytotoxicity Assay

The *in vitro* cytotoxic effect of *A. colubrina* extract was performed on oral fibroblasts cells (HGF-1 ATCC^®^ CRL-2014) and determined by a resazurin fluorometric method (Cell Titer Blue Viability Assay, Promega Corp, Madison, WI). Oral fibroblast cells were cultured in Dulbecco’s modifed Eagle’s medium (DMEM, Lonza, Walkersville, MD) with 10% fetal bovine serum (FBS Gibco, Invitrogen, Waltham, MA) at 37°C in 5% CO_2_. Fibroblast cells (1 × 10^5^°cells/mL) were first seeded in each well of a 24-well plate in DMEM with 10% FBS, and the plates were incubated for 24 h at 37°C in 5% CO2. The *A. colubrina* extract was diluted in DMSO 1%, with final concentration inside the wells of 0.1%, and then added to the cultured cells wells (2,500–0.25 μg/ml). The plates were incubated for 24 h at 37°C in 5% CO_2_. Resazurin (30 µL) was added to each well, and the cells were incubated for 3 h. The fluorescence of the supernatant was read in a microplate reader with excitation of 555 nm, emission of 585 and 570 nm *cut off* (O’Brien et al., 2000).

### Co-Culture Model Fluorescence Microscopy

A co-culture model was conducted by culturing fibroblast cells and *C. albicans* together in a sterile 24-well plate, as adapted by Wong et al. (2014), Oliveira Silva et al. (2018). First, oral fibroblast cells were seeded in DMEM with 10% FBS at 37°C in 5% CO_2_ for 24 h. The medium was then replaced with an inoculum of 5 × 10^3^ to 2.5 × 10^3^ CFU/mL *C. albicans* (MYA 2876) grown in DMEM without FBS. Fibroblast cells and *C. albicans* were treated with 33.28 μg/ml of *A. colubrina* extract. The plate was then incubated at 37°C in 5% CO_2_ for 24 h. The vehicle control tested was 0.1% DMSO and the positive control was Fluconazole (10 μg/ml). The distribution of dead and live fibroblast cells was examined using the viability/cytotoxicity Live/Dead Assay Kit for mammalian cell type (Molecular Devices, Sunnyvale, CA), which contains a mixture of Calcein AM and EthDIII (Ethidium Homodimer III). Calcofluor white (Sigma Aldrich, San Luis, MO) was used to stain *C. albicans*. Fluorescent images of the double staining were captured using fluorescence microscopy (Keyence All-in-One BZ-X810 Fluorescence Microscope, Itasca, IL).

### Co-Culture Model Scanning Electron Microscopy

Co-culture model was conducted by culturing fibroblast cells and *C. albicans* together in a sterile petri dish (Greiner Bio-One North America, Inc. Monroe, NC), following the same protocol above described for co-culture plating and treatments. After the period of incubation, the samples were washed twice with PBS and fixed in glutaraldehyde 3% (v/v) at room temperature for 12 h. The dehydrated cells were submitted to sequential baths of ethanol at concentrations of 50, 70, 90% and absolute ethanol twice, then coated with gold/palladium alloy in a Metalizer (Desk V Denton Vacuum, Moorestown, NJ) and observed using a Scanning Electron Microscope (Zeiss EVO LS10 SEM, Oberkochen, Alemanha) (Bersan et al., 2014; Freires et al., 2014).

### Co-Culture Model Quantitative Real-Time PCR

Following the same protocol above described for co-culture plating, RNA was isolated from fibroblast cells and *C. albicans* after 8 h of treatment with *A. colubrina* extract. The fibroblast cells RNA and *C. albicans* RNA were isolated and purified using RNeasy^®^ Mini Kit (Qiagen, Hilden, Germany) and RiboPure™-Yeast Kit (Invitrogen, ThermoFischer Scientific, Rockford, IL) respectively. SpectraDrop Micro-Volume Starter Kit (Molecular Devices, Sunnyvale, CA) was used to quantify the total RNA extracted. Real-time PCR was conducted by using QuantiFast^®^ SYBR^®^ Green RT-PCR One Step Kit (Qiagen, Hilden, Germany). The *C. albicans* primers for the genes: *Secreted Aspartyl Proteinases-1* (SAP-1), *Phospholipase B-1* (PLB-1), and ACT-1 (*housekeeping*) at 10 µM were used. ACT-1 was the gene used to normalize SAP-1 and PLB-1 genes expression. The following host inflammatory cytokines genes were selected: IL-6 (Qiagen Gene ID#: 3570), IL-8 (Qiagen Gene ID#: 3576); IL-10 (Qiagen Gene ID#: 3587); IL-1β (Qiagen Gene ID#: 3553) and GAPDH (*housekeeping*) (Qiagen Gene ID#: 2597). All data from cytokines genes expression were normalized using the housekeeping gene GAPDH. PCR amplification was performed by using 25 µl reaction mix per well in 0.2 ml 8-Strip PCR tubes. The reactions were conducted in thermocycler (QuantStudio 3 Real Time PCR System, ThermoFischer Scientific, Rockford, IL) at 50°C for 10 min (Reverse Transcription Step); 95°C for 5 min (PCR Initial Activation Step); followed by 40 cycles of 10 s at 95°C (Denaturation Step) and 30 s at 60°C (Annealing/Extension Step). Analysis of relative gene expression was achieved according to the ΔΔCt method (Seleem et al., 2016a; Seleem et al., 2016b; Chen et al., 2018).

### Host Inflammatory Cytokines Analysis Using Luminex

As previously described, co-culture models were performed using fibroblasts cells, *C. albicans* (MYA2876), and the tested groups of *A. colubrina* extract (33.28 μg/ml), positive control (Fluconazole 10 μg/ml) and 0.1% DMSO (vehicle control). After 8 h of incubation, the supernatants of the co-culture were collected, centrifuged for 10 min at 1,000 rpm, and assayed immediately using Human Magnetic Premixed Multi-Analyte Luminex Assay Kit (R&D Systems, Mienneapolis, MN) for secretion of pro-inflammatory cytokines IL-6, IL-8, IL-1β, and anti-inflammatory IL-10. Culture supernatants and cytokine capture bead cocktails were incubated during the overnight period. The samples were then incubated for 1 h with biotin-labeled antibody and for 30 min in a dilution of streptavidin-PE. Data were obtained by Luminex 200 Milliplex System and analyzed with Milliplex Analyst software (Allin et al., 2016).

### Statistical Analysis

Data were expressed as the mean ± SEM using one-way analysis of variance (ANOVA) and Dunnett’s multiple comparison tests in relation to the vehicle, using GraphPad Prism software (version 8.02). Results were considered significant if *p*-values were less than 0.05.

## Results

### Phytochemical Analysis

Standardization of plant inputs for the pharmaceuticals industry plays an important role in product outcomes. The quality of the botanical material, as well as the adequate processing of the fresh material, including drying, transportation, storage, and the use of appropriate and reproducible extraction techniques have a straight outcome on health benefits and economic issues. Previous studies were conducted to determine the best conditions for *A. colubrina* extract preparation (Lima et al., 2014; Rocha et al., 2017; Lima et al., 2018–*data not published*; Maia et al., 2021) The presence of polyphenol content of 53.1% (Lima et al., 2014) and 53.18% (Rocha et al., 2017) gallic acid equivalents; tannins (8.7% catechin equivalents) and flavonoids (0.3% quercetin equivalents) (Lima et al., 2014), compounds with straight relationship with *A. colubrina* extract activity were also recently monitored by GC-MS analysis (Maia et al., 2021). These results, presented in Table 1, suggest the maintenance of the same phytochemical profile of the standardized extract.

**TABLE 1:** Phytochemical compounds identified in the hydroalcoholic bark extract of *A. colubrina* by GC-MS (Maia et al., 2021).

	Compound	RT	RI	Area (%)
1	2-Piperidinecarboxylic acid, 1TMS	8.404	1,267	0.06
2	Butanedioic acid, 2TMS	9.079	1,319	0.02
3	Glyceric acid, 3TMS	9.380	1,343	0.01
4	Isoeugenol, 1TMS	11.065	1,482	0.03
5	Malic acid, 3TMS	11.248	1,498	0.15
6	Erythritol, 4TMS	11.422	1,513	0.01
7	Erythronic acid, 4TMS	12.151	1,574	0.02
8	4-Hydroxybenzoic acid, 2TMS	12.918	1,636	0.02
9	d-Ribose, 2,3,4,5-tetrakis-O-(trimethylsilyl)-, O-methyloxime	13.285	1,664	0.01
10	Xylitol, 5TMS	14.295	1740	0.90
11	Xylitol, 5TMS	14.370	1745	0.1
12	β-D-Xylopyranose, 4TMS	14.510	1755	0.02
13	D-(+)-Galactopyranose, 5TMS	16.390	1884	0.02
14	D-Glucopyranose, 5TMS	16.930	1920	1.37
15	D-Glucopyranose, 5TMS	17.100	1930	0.04
16	D-Mannitol, 6TMS	17.560	1960	0.18
17	Gallic acid, 4TMS	17.800	1975	0.02
18	Palmitic acid, 1TMS	18.965	2047	0.07
19	Myo-inositol, 6TMS	20.220	2,125	0.27
20	Stearic acid, 1TMS	22.210	2,246	0.02
21	Sucrose, 8TMS	29.378	2,705	48.08
22	Catechin, 5TMS	32.458	2,920	0.52

### *In Vitro* Antifungal Activity

MIC and MFC values for *A. colubrina* extract and for the standard antifungal on *Candida* spp. are illustrated in Table 2. For the extract, the MIC were lower than 15.62 μg/ml and the MFC ranged from 250 μg/ml to higher than 2,000 μg/ml, while MIC and MFC of Fluconazole ranged from lower than 1 to 8 μg/ml and from 32 to higher than 128 μg/ml, respectively. The extract demonstrated strong antifungal activity against all *Candida* strains tested, based on the classification proposed by Holetz et al. (2002). According to the parameters stated by Siddiqui et al. (2013), the ratio MFC/MIC found demonstrated a fungistatic effect of the extract against the species tested.

**TABLE 2:** Minimum Inhibitory Concentration (MIC) and Minimum Fungicidal Concentration (MFC) of *A. colubrina* extract against *Candida* strains.

	*A. colubrina*			Fluconazole		
*Candida* strain	MIC (µg/ml)	MFC (µg/ml)	MFC/MIC *ratio*	MIC (µg/ml)	MFC (µg/ml)	MFC/MIC *ratio*
*Candida albicans* ATCC^®^ 90028	<15.62	>2,000	>4	<1	64–128	>4
*Candida albicans* ATCC^®^ MYA-2876	<15.62	2,000	>4	<1	64–128	>4
*Candida glabrata* ATCC^®^ MYA-275	<15.62	>2,000	>4	8	>128	>4
*Candida tropicalis* ATCC^®^ MYA-750	<15.62	250	>4	4	>128	>4
*Candida dubliniensis* ATCC^®^ MYA-646	<15.62	>2,000	>4	<1	32–64	>4

Note: MFC/MIC <4: fungicidal profile/MFC/MIC >4: fungistatic profile.

### Biofilm Inhibition

**FIGURE 1:**
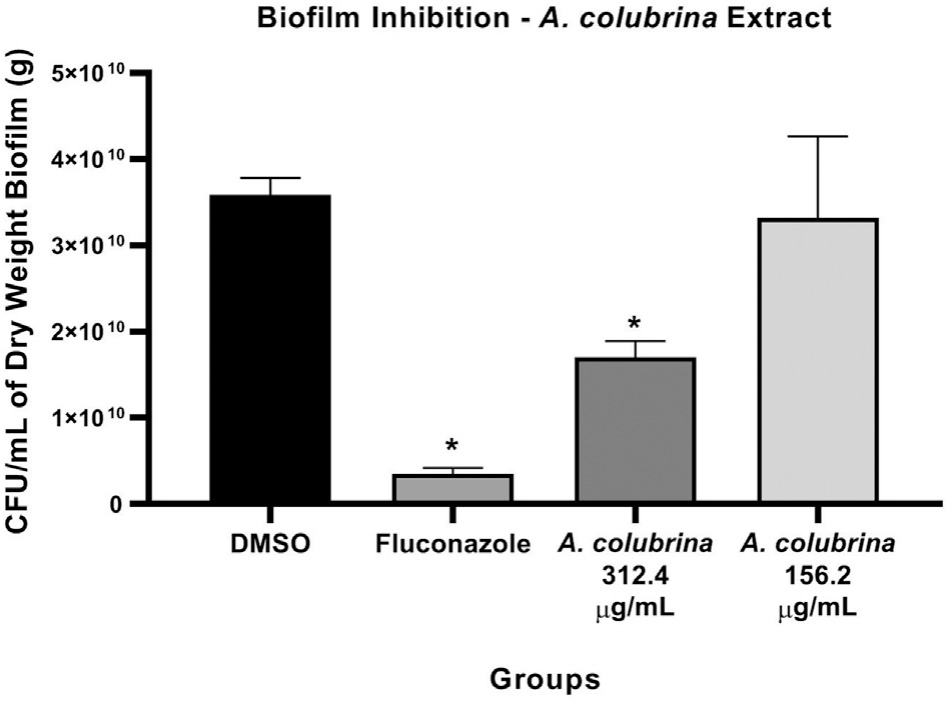
Fungal viability of *C. albicans* MYA 2876 72-hours-biofilm expressed in CFU/ml/grams of dry weight after treatment with *A. colubrina* extract (312.5 μg/ml–20 × 15.62 μg/ml; 156.2 μg/ml–10 × 15.62 μg/ml). DMSO 0.1%: vehicle control; Fluconazole (10 μg/ml): positive control. Values shown with asterisk (*) are statistically significant when compared to the vehicle control (*p* < 0.05).

Biofilm assay showed that the treatments with *A. colubrina* extract at 312.4 μg/ml, equivalent to 20 × 15.62 μg/ml for *C. albicans* MYA 2876, and with Fluconazole at 10 μg/ml (10MIC) had significant reduction (*p* < 0.05) in fungal load, expressed as CFU/ml/g of biofilm dry weight, in comparison to the vehicle control group (DMSO 0.1%) (Figure 1).

### Proteinase and Phospholipase Enzymes Secretion

**FIGURE 2:**
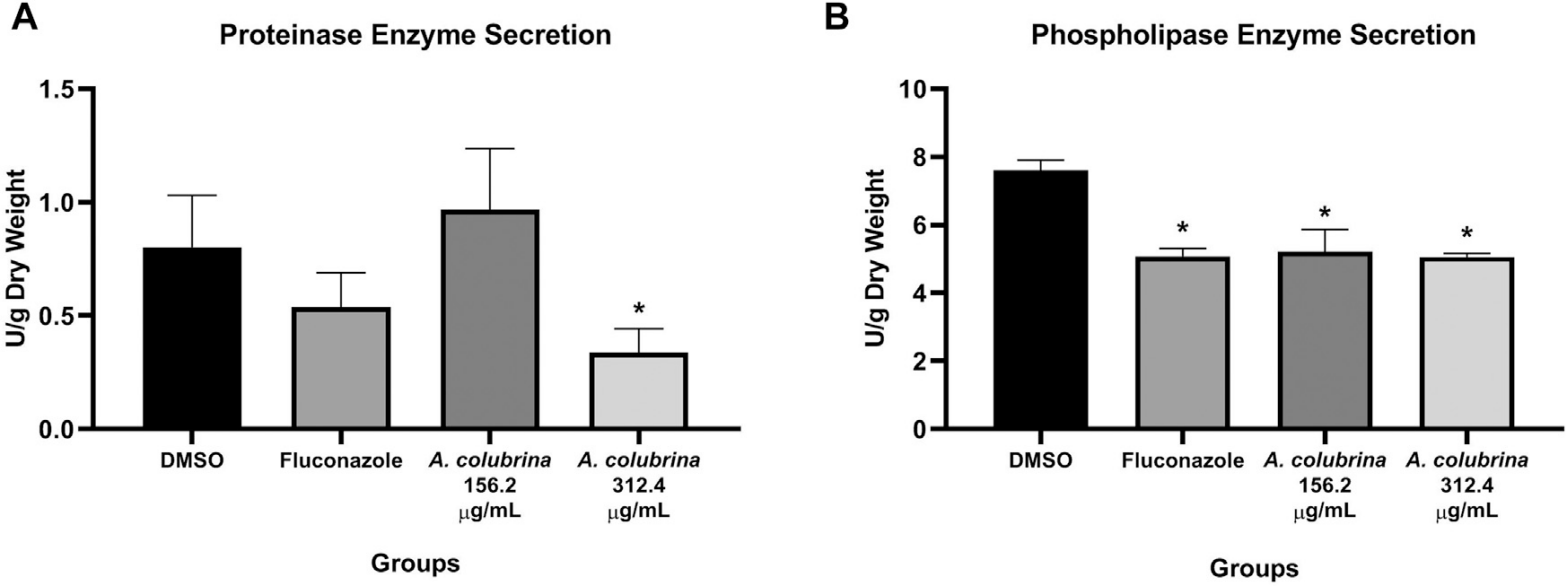
*C. albicans* MYA 2876 72-hours-biofilm proteolytic enzymes secretion expressed in U/grams of dry weight after treatment with *A. colubrina* extract (312.5 μg/ml–20 × 15.62 μg/ml; 156.2 μg/ml–10 × 15.62 μg/ml). **(A)** Proteinase activity; **(B)** Phospholipase activity. DMSO 0.1%: vehicle control; Fluconazole (10 μg/ml): positive control. Values shown with asterisk (*) are statistically significant when compared to the vehicle control (*p* < 0.05).

Phospholipase enzyme activity was significant reduced (*p* < 0.05) after the treatments with *A. colubrina* at 156.2 μg/ml (10 × 15.62 μg/ml) and 312.4 μg/ml (20 × 15.62 μg/ml), when compared to the vehicle (DMSO 0.1%). On the other hand, the proteinase enzyme activity was significant decreased (*p* < 0.05) by the extract only at 312.4 μg/ml (20 × 15.62 μg/ml) in comparison to the vehicle control group (Figures 2A,B).

### Co-Culture Model of Fibroblasts and *C. albicans*

#### Cytotoxicity

**FIGURE 3:**
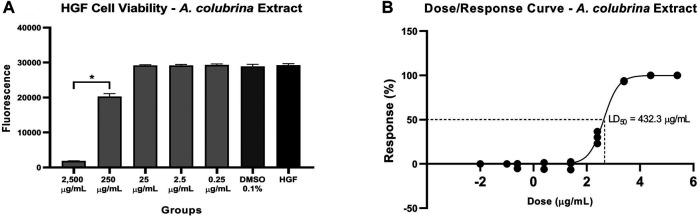
**(A)** Cytotoxicity effect of *A. colubrina* extract (2,500 μg/ml–0.25 μg/ml) on human gingival fibroblasts after 24 h of treatment, **(B)** LD_50_ = 432.3 μg/ml. HGF: only cells; DMSO 0.1%: vehicle control (Values shown with asterisk (*) are statistically significant when compared to the vehicle control (*p* < 0.05).

The *A. colubrina* extract presented LD_50_ of 432.3 μg/ml and a non-toxic profile on gingival fibroblast cell culture up to a concentration of 250 μg/ml, with cell viability remaining higher than 70%, when compared to the vehicle and the cell control groups, as shown in Figure 3.

#### Proteolytic Enzymes Gene Expression

**FIGURE 4:**
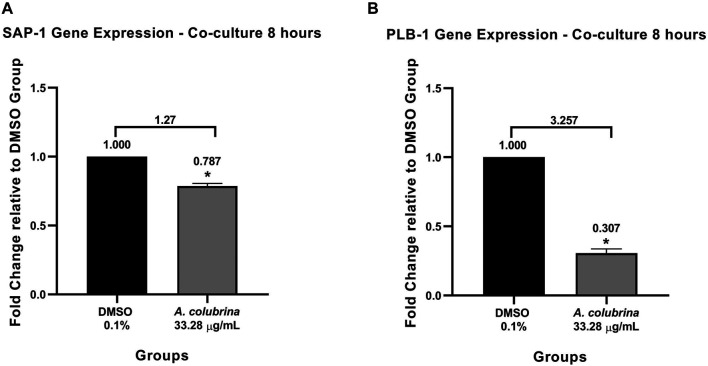
Real-time quantitative information about relative gene expression of **(A)** SAP-1 and **(B)** PLB-1 after 8 h of treatment with *A. colubrina* extract treatment (33.28 μg/ml) in human gingival fibroblasts infected by *C. albicans*. DMSO 0.1%: vehicle control. Values shown with asterisk (*) are statistically significant when compared to the vehicle control (*p* < 0.05).

Similar to the profile obtained from the biofilm proteinase and phospholipase assays, the gene expression of SAP-1 and PLB-1 secreted by *C. albicans* MYA 2876, grown as immature biofilm in a co-culture model, was significantly down-regulated (*p* < 0.05) after the exposure to *A. colubrina* extract at 33.28 μg/ml, when compared to the vehicle control group (Figures 4A,B).

#### Fluorescence Microscopy

**FIGURE 5:**
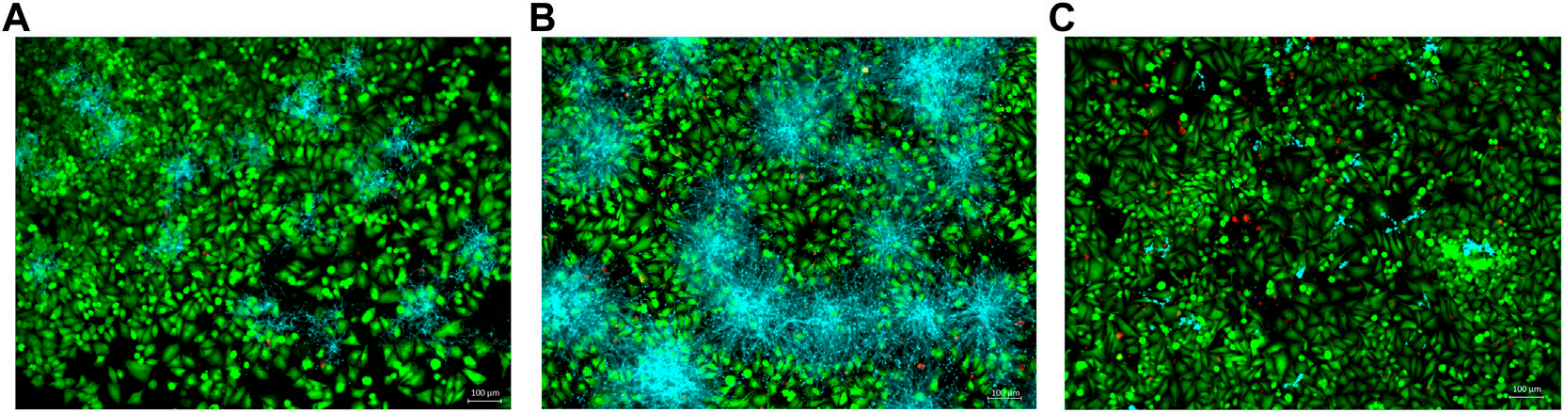
Co-culture fluorescence microscopy after 24 h of treatment with *A. colubrina* extract. **(A)** *A. colubrina* extract (33.28 μg/ml); **(B)** Vehicle control (DMSO 0.1%) and **(C)** Positive control (Fluconazole–10 μg/ml). Scale bar set at 100 μm at 100x magnification power.

In the co-culture model of fibroblasts coexisting with *C. albicans*, samples treated with *A. colubrina* extract showed a considerable decrease in *Candida* growth distribution in comparison with the vehicle control, as indicated by the sparse and less dense accumulation of *C. albicans* (blue color) among viable fibroblast cells (green color) in fluorescent images. In addition, fibroblasts viability was not significantly affected by the treatment with the extract, since there was no significant increase in the dead fibroblast population, indicated by the red fluorescent color, suggesting that *A. colubrina* was effective against *C. albicans* with minimal effects or toxicity on fibroblast cells (Figures 5A–C).

#### Scanning Electron Microscopy

**FIGURE 6:**
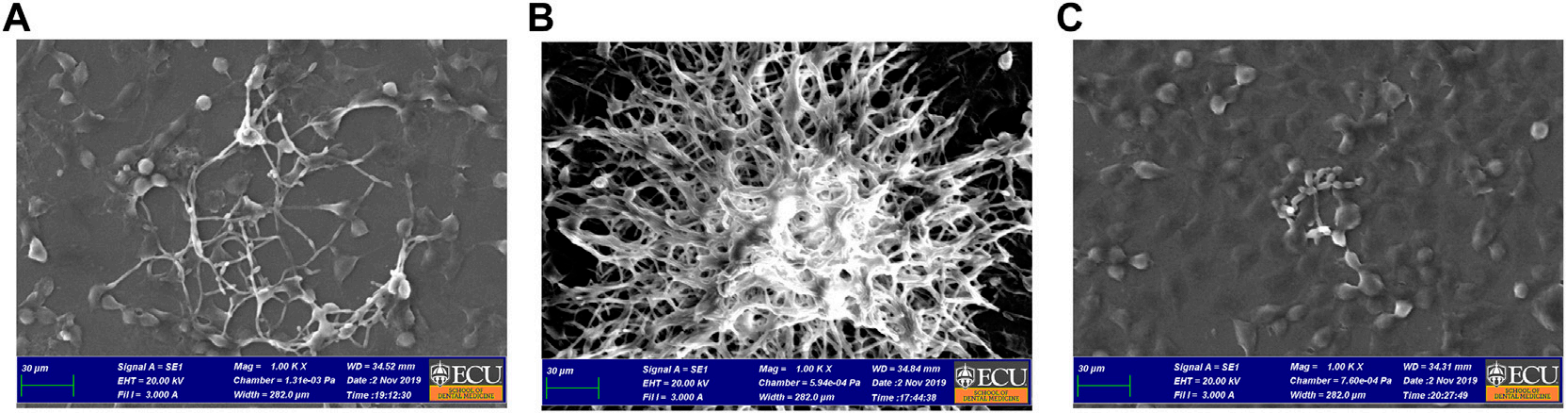
Co-culture SEM after 24 h of treatment with *A. colubrina* extract. **(A)** *A. colubrina* extract (33.28 μg/ml); **(B)** Vehicle control (DMSO 0.1%) and **(C)** positive control (Fluconazole–10 μg/ml). Scale bar set at 30 μm at 1,000x magnification power.

As seen in Figures 6A–C, the pattern was similar to the arrangement observed on Fluorescence Microscopy. The SEM showed density reduction of *C. albicans* biofilm, and de-structuring of hyphae morphology, in comparison to the vehicle control group.

#### Pro and Anti-inflammatory Cytokines Gene Expression

**FIGURE 7:**
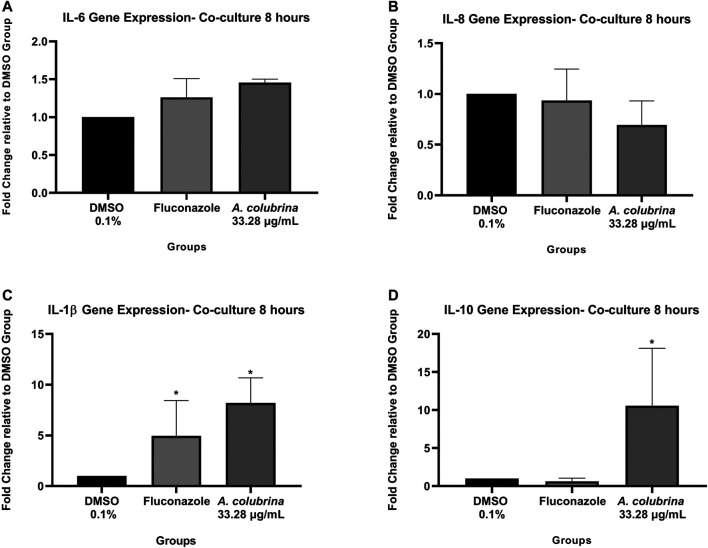
Real-time quantitative information about relative gene expression of **(A)** IL-6; **(B)** IL-8; **(C)** IL-1β; and **(D)** IL-10 of human gingival fibroblasts after 8 h of infection by *C. albicans* and treatment with *A. colubrina* extract (33.28 μg/ml). DMSO 0.1%: vehicle control; Fluconazole (10 μg/ml): positive control. Values are shown as the fold-change relative to the vehicle control group. Values shown with asterisk (*) are statistically significant when compared to the vehicle control (*p* < 0.05).

The *A. colubrina* extract at 33.28 μg/ml presented a modulatory effect on the gene expression of host inflammatory cytokines in a co-culture model. The gene expression of IL-6, IL-1β, and IL-10 were up-regulated after host cells exposure to the extract, while IL-8 gene expression was down-regulated. However, the *A. colubrina* concentration tested showed no statistical difference (*p* > 0.05) on the gene expression of the host cytokines in comparison to the vehicle control group (Figures 7A–D).

#### Pro and Anti-inflammatory Cytokines Secretion

**FIGURE 8:**
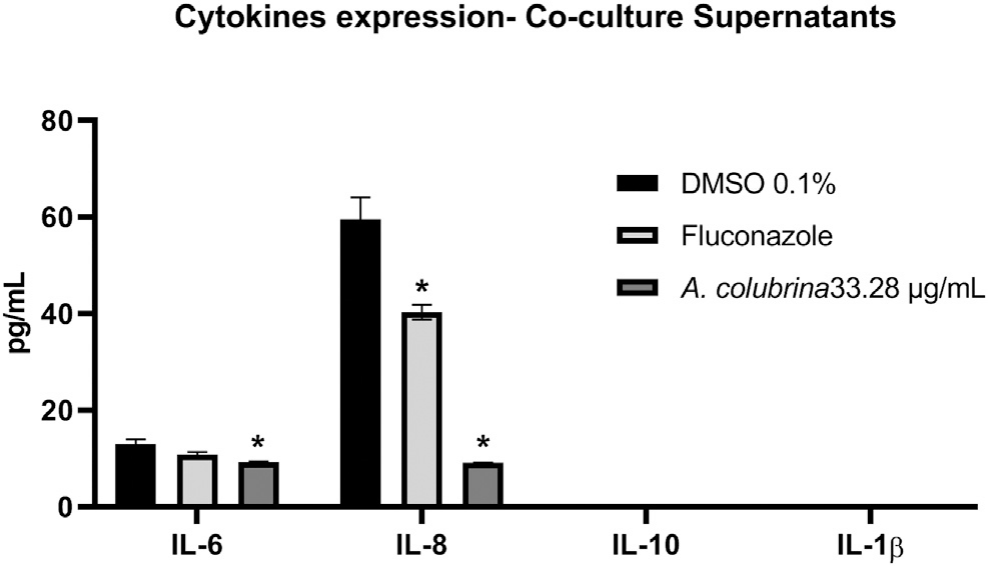
Pro and anti-inflammatory cytokines expression of IL-6, IL-8, IL-10 e IL-1β by human gingival fibroblasts after 8 h of infection with *C. albicans* and treatment with *A. colubrina* extract (33.28 μg/ml). HGF: only cells; DMSO 0.1%: vehicle control; Fluconazole (10 μg/ml): positive control. Values shown with asterisk (*) are statistically significant when compared to the vehicle control (*p* < 0.05).

Co-culture supernatants were assessed for expression of pro-inflammatory IL-6, IL-8, and IL-1β as well as anti-inflammatory cytokine IL-10, following treatments with *A. colubrina* extract (33.28 μg/ml). The extract has resulted in a significant reduction (*p* < 0.05) in the expression of IL-6 and IL-8 when compared to the vehicle control group. On the other hand, there was no detection of modulatory effect on the expression of IL-1β and IL-10 cytokines upon treatment with the extract (Figure 8).

## Discussion

Recently, the traditional therapeutic use of *A. colubrina* by popular communities in Brazilian culture has encouraged comprehensive studies about its biological properties to be conducted (Damascena et al., 2014; Lima et al., 2014; Barreto et al., 2016; Mota et al., 2017; Guarneire et al., 2019; Silva et al., 2019; Cardoso-Junior et al., 2020; Maia et al., 2021). In this study, we evaluated the *A. colubrina* extract effects on virulence factor of *C. albicans* and its modulatory effects on host immune response during the fungal infection.

In a previous study, we evaluated the phytochemical composition of the *A. colubrina* extract (Lima et al., 2014), in which was found that the extract presents in its composition a high total polyphenol content (53.18% gallic acid equivalents); tannins (8.77% catechin equivalents) and flavonoids (0.28% quercetin equivalents). This chemical profile seems to support the biological activities evaluated here and discussed ahead, since these classes of compounds are related to a wide range of properties, including antifungal (Ramirez et al., 2013; Kanchanapiboon et al., 2020) and immune response modulatory effects (Fu et al., 2013; Ji et al., 2019; Cardoso Junior et al., 2020).

*A*. *colubrina* extract has been reported to present *in vitro* antifungal properties against *Candida* spp. and anti-biofilm properties, mainly on *C. albicans* biofilms (Lima et al., 2014; Silva et al., 2019). The susceptibility assay from our study showed a strong anti-*Candida* effect (Holetz et al., 2002) of *A. colubrina*, exerting a fungistatic profile (Siddiqui et al., 2013) over all the strains tested in planktonic form. *Candida* spp. is not only an important component of oral microbiota, living as a common commensal in immunocompetent individuals (Qin et al., 2016), but also display an important role in shaping the oral microbiome (Xu and Dongari-Bagtzoglou, 2015; Bertolini et al., 2019). Thus, total elimination of yeast from the body is neither desirable nor feasible (Bhattacharya et al., 2020) and its growth inhibition instead its elimination might be positive regarding the infection control, by preventing the rise of pathogenic strains that could lead to more severe infections and antifungal resistance (Ford et al., 2015).

One of the major virulence factors related to *C. albicans* pathogenesis, with significant clinical implications, resides in its ability to form biofilms (Fox et al., 2015; Wall et al., 2019). In oral cavity, hyphae formation and adherence to oral epithelial cells and other abiotic surfaces, such as dentures, leads to the development of this structured community of surface-associated microbial populations embedded in an extracellular matrix (Ghannoum et al., 2015; Hirota et al., 2017). In our study, biofilms treated with *A. colubrina* at 312.4 μg/ml (20 × 15.62 μg/ml) showed significant decrease in fungal viability in comparison to the vehicle group, by reducing the CFU/mL/g parameter and altering the biofilm composition and structure integrity. For this biofilm assay model, we used *C. albicans* MYA 2876, considering the MFC result of this strain after the treatment with the extract. In addition, higher concentrations of *A. colubrina*, equivalent to 156.2 μg/ml (10 × 15.62 μg/ml) and 312.4 μg/ml (20 × 15.62 μg/ml), were used against *C. albicans* MYA 2876 strain, due to biofilm structured and stable environment, which is tolerant to antifungal agents’ diffusion (Tsui et al., 2016).

Once biofilm is established, the expression of *C. albicans* virulence factors increases (Jabra-Rizk, 2011) also giving rise to the release of extracellular hydrolytic enzymes by the biofilms into the local environment, contributing to candidiasis progression (Vila et al., 2020). Most notable of the secreted enzymes frequently implicated in the pathogenicity of *C. albicans* are Secreted Aspartyl Proteinases (SAPs) and Secreted Phospholipases (PLs), which are involved in host tissue invasion, nutrient acquisition, immune evasion, and organ damage (Jabra-Riks, 2011; Sorgo et al., 2013; Swidergall and Filler, 2017). In order to evaluate the modulating effect of *A. colubrina* extract over this critical virulence factor, we demonstrated that phospholipase enzyme activities was significant reduced using the extract both at 156.2 μg/ml (10 × 15.62 μg/ml) and 312.4 μg/ml (20 × 15.62 μg/ml) concentrations, while proteinase activity was decreased only by the highest concentration. These results suggest that both antifungal and anti-biofilm activities of *A. colubrina* could involve the inhibition of proteolytic enzymes expression as mechanism of action, considering that during *C. albicans* penetration, these enzymes affect epithelial junctions and enable degradation of cell membrane components, facilitating fungal adhesion and biofilm formation to both oral epithelium or abiotic surfaces (Naglik et al., 2011).

To confirm the results obtained over proteolytic enzyme secretion, we assessed gene expression of SAP-1 PLB-1) after treatment with *A. colubrina* at 33.28 μg/ml on co-culture models of immature biofilm of *C. albicans* coexisting with fibroblasts. We demonstrated a significant down-regulation on the gene expression of both enzymes in comparison to the vehicle group. These findings are consistent to the enzymatic secretion results, considering that fungal infections usually present a higher gene expression of SAPs and PLs. Proteinases were shown to induce degradation of a variety of host factors, such as E-cadheryn, present in epithelial cell junction, increasing the hyphae capacity for colonization and penetration into host tissues, and factors involved in the innate and adaptive immune responses, allowing *C. albicans* to overcome the host immune defenses (Kumar et al., 2017). On the other hand, phospholipases expression displays an active role in epithelial adherence and tissue invasion, since their catalytic action results in disruption of epithelial cell membrane allowing the penetration of yeasts into host cell cytoplasm (Sanitá et al., 2014). Thus, our results suggest that the *A. colubrina* can act by interfering with yeast invasion mechanisms, which could prevent the development of candidiasis.

The cytotoxicity assay was conducted on human gingival fibroblasts (LD_50_ = 432.3 μg/ml) in order to verify the therapeutic safety level previously, regarding further *in vivo* and human clinical researches. *A. colubrina* extract maintained the cell viability higher than 70% at concentrations up to 250 μg/ml, representing a relatively low cytotoxic activity. This finding is consistent with results recently reported, which verified the low toxicity of the bark extract of *A. colubrina* on human cells lines, such macrophages (Silva et al., 2019; Cardoso-Junior et al., 2020), keratinocytes and also on tumoral cell strains (Lima et al., 2014). These data corroborate to the results found in this study related to the low toxic potential effect of this product, shown to be pharmacological safe *in vitro*, by maintaining the cell viability.

Considering the purpose to evaluate the regulating effects of *A. colubrina* extract on *C. albicans*-host interaction, we used a co-culture model to provide comprehensions about the complex system of an immature *C. albicans* biofilm coexisting with fibroblast cells incubated with the tested extract. Recent studies had been conducted in order to evaluate *in vitro* the modulatory effects of natural compounds on the interaction between *C. albicans* and the host using a co-culture model (Seleem et al., 2016a; Seleem et al., 2016b; Chen et al., 2018). Moreover, co-cultures have demonstrated to be an effective tool to stimulate physiological conditions and induce interactions between the cells that triggers important host response pathways (Serrano et al., 2017).

Microscope images obtained from the co-culture model were helpful to evaluate qualitatively the distribution of *C. albicans* and fibroblasts in response to *A. colubrina* and the controls groups. The pattern imaging observed through fluorescence microscopy demonstrated a considerable reduction in *C. albicans* growth without affecting significantly the fibroblasts viability, showing a strong antifungal effect with minimal toxicity. Similarly, the arrangement observed on co-culture SEM imaging in a higher magnification and topographic view, showed significant alterations of the biofilm structure treated with *A. colubrina*. When compared to the vehicle group, it is possible to observe structural alterations, such as notable decrease of the density biofilm and de-structuring of the hyphae morphology in the areas where the extract was able to penetrate. In addition, hyphae decreased, indicating a potential reduction of the biofilm virulence, since the formation of hyphae is associated to tissue invasion.

Host immune defense against *C. albicans* infection requires a wide range of complex molecular mechanisms involving the recognition of fungal cell wall components, the activation of host immune cell signaling cascades and the release of cytokines and chemokines (Brown et al., 2012; Pappas et al., 2018). Some reviews were recently published regarding the interplay between *C. albicans* and host cells and the immune response during *C. albicans* mucosal infection (Höfs et al., 2016; Naglik et al., 2017; Nikou et al., 2019; Swidergall, 2019; Vila et al., 2020), serving as a guidelines to improve our understanding that relies on this complex interaction.

Therefore, to evaluate the modulatory activity of *A. colubrina* on the host pro-inflammatory response during the *Candida* infection, we employed a transcriptomic approach in conjunction with a proteomic profiling, regarding the analysis for the host cells’ gene expression and the release of pro-inflammatory and anti-inflammatory cytokines. We choose a panel of inflammatory markers according to the host immune response mechanisms upon *Candida* recognition, considering the activation of transmembrane Toll Like Receptors (TRLs) (Salvatori et al., 2016; Pappas et al., 2018), which triggers signaling pathways and results at the release of pro-inflammatory cytokines, mainly IL-1α, IL1β, IL-6, IL-8, and CCL5 (RANTES) by the host cells (Hebecker et al., 2014; Verma et al., 2017; Verma et al., 2018).

The assessment of gene expression demonstrated that *A. colubrina* affects the expression of host inflammatory cytokines by down-regulating IL-8 gene expression and up-regulating IL-6, IL-1β, and IL-10 gene expression. However, there was no statistical difference when compared to vehicle control group. On the other hand, the analysis for expression of host pro-inflammatory and anti-inflammatory cytokines was found significant reduction on the secretion of pro-inflammatory cytokines IL-6 and IL-8, suggesting a modulatory role of *A. colubrina* on the host pro-inflammatory response, which could help to eradicate the fungal infection. A possible reason for the differences between gene expression and production profile of IL-1β, IL-6 an IL-10 is based on a negative feedback mechanism modulating gene expression. Cytokines gene expression may play an important role in regulating the release of them. However, as indicated by the significant reduction in expression of these molecules, there may be a negative feedback inhibition mechanism involving the transcription and translation processes, which regulates their respective gene expression. However, this hypothesis needs to be confirmed through further molecular studies.

As mentioned before, IL-6 and IL-8 play a crucial role in innate immune response. While IL-8 is a known neutrophil recruiter from the circulating blood to the site of infection (Höffs et al., 2016), IL-6 is related to immune response to microorganisms and its expression is directly influenced by the secretion of other pro-inflammatory cytokines, especially IL-1 (Tanaka et al., 2014). Some reports state that *C. albicans* infection is crucial for the modulation and release of IL-1α, IL-6, and IL-8, promoting a higher production of these cytokines (Feller et al., 2014; Figueira et al., 2020; Mo et al., 2020). In this regard, our findings suggest that *A. colubrina* might reduce pro-inflammatory IL-6 and IL-8 cytokines expression during the fungal infection, due its strong antifungal activity, which modulates some putative virulence factors of the *C. albicans*, such as biofilm formation and proteolytic enzyme secretion, reducing the fungus pathogenicity. In addition, the low cytotoxicity of the extract on the host cells does not induce an inflammatory response.

This is the first study reporting the effects of *Anadenanthera colubrina* (Vell.) Brenan on *Candida albicans*-Host interaction. *A. colubrina* extract demonstrated anti-*Candida*, antibiofilm and anti-proteolytic enzyme activities against *C. albicans*, with relatively low cytotoxicity to the host cells. Furthermore, presented modulatory effects on the host immune response, as indicated by its regulation of IL-6 and IL-8 pro-inflammatory cytokines secretion. In this regard, *A. colubrina* could be considered as a potential source for an anti-*Candida* formulation. Further investigations about the regulation activity of *A. colubrina* on others *C. albicans* key virulence factors, including expression of cell surface adhesins, proteolytic degradation of host immune factors and invasion and destruction of host tissue mechanisms, must to be conducted in order to reinforce and establish the extract effects over molecular and signaling pathways in *Candida* pathogenesis.

## Data Availability

The raw data supporting the conclusions of this article will be made available by the authors, without undue reservation, to any qualified researcher.
